# Health factors associated with cognitive frailty in older adults living in the community

**DOI:** 10.3389/fnagi.2023.1232460

**Published:** 2023-09-18

**Authors:** Juan Corral-Pérez, Cristina Casals, Laura Ávila-Cabeza-de-Vaca, Andrea González-Mariscal, Ildefonsa Martínez-Zaragoza, Francisca Villa-Estrada, Remedios Reina-Campos, María Á. Vázquez-Sánchez

**Affiliations:** ^1^ExPhy Research Group, Department of Physical Education, Instituto de Investigación e Innovación Biomédica de Cádiz (INiBICA), Universidad de Cádiz, Cádiz, Spain; ^2^Puerta Blanca Health Centre, Malaga-Guadalhorce Health District, Málaga, Spain; ^3^Trinidad Jesús Cautivo Health Centre, Malaga-Guadalhorce Health District, Málaga, Spain; ^4^Huelin Health Centre, Malaga-Guadalhorce Health District, Málaga, Spain; ^5^Department of Nursing, Faculty of Health Sciences, University of Malaga, Málaga, Spain; ^6^PASOS Research Group, UMA REDIAS Network of Law and Artificial Intelligence Applied to Health and Biotechnology, University of Malaga, Málaga, Spain

**Keywords:** physical activity, physical function, accelerometer, quality of life, malnutrition risk, cognitive impairment, aging, frail adults

## Abstract

**Introduction:**

This study aims to investigate the health factors associated with cognitive frailty in frail and pre-frail older adults living in the community.

**Methods:**

A total of 233 older adults meeting Fried’s criteria for pre-frailty or frailty were included. Cognitive status was evaluated using the Short Portable Mental Status Questionnaire. Health factors encompassed nutritional status (evaluated using the Mini Nutritional Assessment tool, body mass index, and waist, arm, and leg circumferences), physical function (assessed with the Short Physical Performance Battery), quality of life (measured with the total index of the EuroQoL 5-Dimension 5-Level questionnaire - EQoL-Index -, and the Visual-Analogue Scale - QoL-VAS - for today’s health state), as well as sleep, physical activity, and inactivity estimated through wrist-worn accelerometers. Multivariable logistic regression analyses were conducted to identify potential predictors of cognitive frailty, considering age as a confounding factor.

**Results:**

Cognitive frail participants exhibited advanced age, heightened self-reported exhaustion, diminished overall physical performance, reduced leg perimeter, decreased engagement in moderate-to-vigorous physical activity, and higher levels of inactivity (all p<0.05). However, after adjusting for age, only QoL-VAS emerged as a cognitive frailty risk factor (Odds ratio: 1.024), while the EQoL-Index, calf perimeter, and levels of moderate-to-vigorous physical activity were identified as protective factors (Odds ratios: 0.025, 0.929, and 0.973, respectively).

**Discussion:**

This study highlights the complex relationship between non-modifiable factors such as age, and modifiable factors including quality of life, nutritional status, and physical activity in the development of cognitive frailty among older adults with a frailty phenotype living in the community.

## Graphical abstract

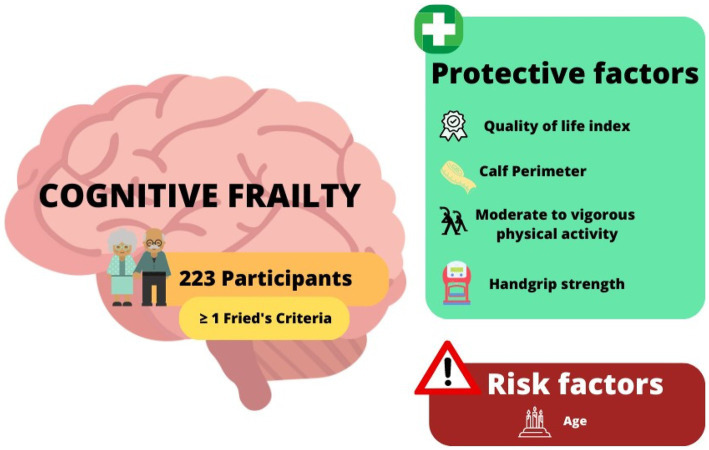

## 1. Introduction

As the aging population continues to grow, frailty remains a prevalent geriatric syndrome among older adults living in the community, thus increasing awareness of its associated risk factors within this population (Clegg et al., 2013; Ofori-Asenso et al., 2019). Frailty is characterized by its multifaceted origin (Pilotto et al., 2020). While a consensus on its precise definition remains elusive, it can be conceptualized as an age-related disruption in the harmonious interplay across various dimensions, including genetic, biological, functional, cognitive, psychological, and socio-economic aspects (Pilotto et al., 2020).

Given this complex nature, the establishment of a universally accepted standard instrument for frailty assessment is currently lacking (Roopsawang et al., 2022). However, the adoption of a multidimensional approach to frailty holds promise for both preventing its onset and ameliorating associated adverse health outcomes (Wleklik et al., 2020). Consequently, although one of the most commonly used frailty assessment tool is the one proposed by Fried et al. (2001), it primarily emphasizes physical frailty, thus highlighting the need for focused attention on the cognitive domain.

The operational definition of cognitive frailty pertains to the concurrent presence of physical frailty and mild cognitive impairment in older individuals, excluding Alzheimer’s disease and various forms of dementia (Kelaiditi et al., 2013). Currently, cognitive frailty is classified into two subtypes: reversible and potentially reversible (Sugimoto et al., 2018). Reversible cognitive frailty involves the simultaneous existence of physical frailty or pre-frailty and subjective cognitive decline or pre-mild cognitive impairment, whereas potentially reversible cognitive frailty is characterized by the coexistence of physical frailty or pre-frailty and mild cognitive impairment (Sugimoto et al., 2018).

Cognitive frailty is associated with an elevated risk of adverse health outcomes, including depression, compromised quality of life, dementia, falls, hospitalization, dysfunction, and even premature mortality (Panza et al., 2018; Sugimoto et al., 2022; Zou et al., 2023). The domains of physical and cognitive frailty are thought to be interconnected (Tang et al., 2023). Notably, cognitive frailty is linked to a decline in executive function (Sugimoto et al., 2022) and limitations in instrumental activities of daily living (Shimada et al., 2016), positioning cognitive frailty as a contributor to the motoric cognitive risk syndrome.

This syndrome is characterized by the coexistence of subjective cognitive complaints and a slow gait, in the absence of concurrent dementia or mobility disability (Verghese et al., 2013; Sugimoto et al., 2018). Various investigations into cognitive frailty have emphasized motor deterioration and gait variables, highlighting the need for specific motor performance assessment within the context of cognitive frailty (Facal et al., 2019, 2021). In this regard, wearable sensors have been proposed to estimate daily levels of physical activity (Zhou et al., 2019; Razjouyan et al., 2020), and accelerometer-based measurements could also be of interest.

Despite recent advances in understanding cognitive frailty, there is still much to be explored. Limited evidence exists regarding the incfluence of health-related factors in older adults which are typically integrated into the comprehensive geriatric assessment, such as mobility, nutritional status, sleep behavior, and physical activity, on cognitive frailty (Pilotto et al., 2020). This knowledge gap underscores the necessity for further research to identify potentially modifiable risk factors and develop targeted interventions aimed at preventing or delaying the onset of cognitive frailty in older adults (Facal et al., 2021; Zhang et al., 2022).

For all of these reasons, this study aims to investigate the health factors associated with cognitive frailty in frail and pre-frail older adults residing in the community. The health factors under consideration include various sociodemographic characteristics, Fried’s frailty components, physical function, quality of life, nutritional status, sleep beaviour, and daily levels of physical activity and inactivity.

## 2. Materials and methods

### 2.1. Design

The current cross-sectional study was carried out in the provinces of Cádiz and Málaga, located in Spain, during the period between March and December 2022. The Ethics Committee of Provincial Research of Málaga approved the study (reference code FRAGSALUD, date 31/01/2019), and all actions adhered to the principles stated in the Declaration of Helsinki for human research. Individuals who indicated their willingness to participate in the study were provided with a document outlining all procedures and potential risks and an informed consent form. Before the commencement of the study, participants were required to affix their signatures on both documents.

A total of 233 older individuals from Spain were included in the study, with an average age of 74.8 ± 6.4 years. To meet the eligibility criteria for participation, participants had to fulfill the following requirements: (i) age 65 years or older, and (ii) presentation of at least one component of Fried’s frailty phenotype. Individuals who were institutionalized or displayed symptoms of dementia were excluded from the study.

Upon inclusion in the study, participants were asked to provide socio-demographic details, including age, sex, marital status, housing conditions, educational attainment, daily medication usage, number of falls in the preceding year, use of walking aids, and approximate monthly income.

### 2.2. Cognitive frailty

The participant’s frailty status was assessed using Fried’s frailty phenotype (Fried et al., 2001), including five domains: (i) unintentional weight loss, (ii) self-reported exhaustion and fatigue, (iii) low weekly physical activity estimated through the short version of the Minnesota Leisure Time Activity Questionnaire (Ruiz Comellas et al., 2012), (iv) low gait speed measured by the 4.57-meter gait test, and (v) low handgrip strength assessed by dynamometer. Individuals meeting one or two of these criteria were classified as pre-frail, while those fulfilling three or more criteria were classified as frail (Fried et al., 2001).

The cognitive status of the participants was evaluated using the Short Portable Mental Status Questionnaire (Pfeiffer, 1975), which has demonstrated good validity and reliability in the Spanish population (Martínez de la Iglesiaa et al., 2001). This test is widely used due to its brevity and ease of administration, making it a rapid screening tool. Comprising 10 questions, it evaluates short-and long-term memory, orientation, and the capacity to perform serial mathematical tasks. A point was awarded for each question that a participant answered incorrectly. Accumulating three or more points (errors) indicates the manifestation of subjective cognitive decline, which could suggest the presence of cognitive impairment. An additional error is permitted in the scoring for individuals with a primary education or lower, while one less error is allowed for those with education beyond the high school level. Participants who met at least one Fried’s frailty criteria and scored higher than three on the Short Portable Mental Status Questionnaire were categorized into the cognitive frailty group.

### 2.3. Nutritional status

The Mini Nutritional Assessment (MNA) was employed to identify participants at nutritional risk (Rubenstein et al., 2001). The MNA contains six items assessing weight loss, body mass index (BMI), illness or stress, mobility, depression or dementia, and loss of appetite. The MNA has been validated for use in populations aged over 65 (Salvà Casanovas, 2012). A MNA score of ≤11 indicates malnutrition or risk of malnutrition. The MNA demonstrated a sensitivity of 97.9%, specificity of 100%, and diagnostic accuracy of 98.7% in predicting malnutrition (Rubenstein et al., 2001).

Participant’s body mass was measured using a digital scale (Omron Medizintechnik, Mannheim, Germany) after removing footwear, heavy clothing, and accessories. To determine body height, participants stood on the Frankfort plane and exhaled normally while using a stature-measuring instrument (SECA 225, Hamburg, Germany). The BMI was calculated by dividing weight (in kilograms) by the square of height (in meters). Waist, arm, and leg circumferences were measured using a non-extensible metallic tape (Lufkin W606PM, Washington, United States) at their narrowest (waist circumference) and longest (arm and leg circumferences) points.

### 2.4. Physical function

The Short Physical Performance Battery (SPPB) was utilized to assess the physical function of the participants, which is a valid and reliable tool for older individuals (Pavasini et al., 2016). The SPPB evaluates three physical domains: (i) balance, including three tests (side-by-side, semi-tandem, and tandem stands), (ii) gait speed over a 4-meter distance, and (iii) lower body performance assessed through the five-repetition sit-to-stand test. Participants’ performance in each domain was compared to normative data and scored between 0 and 4 points. A score of 0 was assigned to participants unable to complete the test. The final score ranged from 0 (highly dependent) to 12 (totally independent).

### 2.5. Quality of life

The EuroQoL 5-Dimension 5-Level (EQ-5D-5L) questionnaire was used to evaluate the health-related quality of life of the participants (Herdman et al., 2011). The EQ-5D-5L is a widely utilized tool comprising five dimensions: mobility, self-care, usual activities, pain/discomfort, and anxiety/depression. Whitin each dimension, participants rate their health status on a Likert scale ranging from no problems (score of 1) to extreme problems (score of 5). The scores from these five dimensions are then combined into a five-digit number representing the participant’s health state. A formula is applied to convert this health state into a single index score (EQoL-Index) that ranges from 0 to 1, with 1 indicating the best possible health status (Oppe et al., 2014). The Visual-Analogue Scale (QoL-VAS) ranges from 0 to 100 where participants rate their overall health status today, with 100 indicating the best possible health. The EQ-5D-5L has been validated in many countries, including Spain (Hernandez et al., 2018), and has shown excellent psychometric properties.

### 2.6. Sleep behavior, physical activity, and inactivity

The GENEActiv wrist-worn accelerometer was used to assess daily sleep behavior, physical activity, and inactivity. Participants were instructed to wear the accelerometer on their non-dominant wrist for a minimum of six consecutive days. Valid results were considered for participants with a wear time of 16 h or more per day, across at least 4 days, including a minimum of three weekdays and one weekend day. The accelerometers were set to operate at a frequency of 60 Hz, and the GENEActiv software (version 3.3) was used to collect the unprocessed data. All unprocessed data files were stored on the University of Malaga servers and were analyzed using the open-source GGIR software (version 2.5-0) with R-package (R CoreTeam, Vienna, Austria). This R-package was applied to mitigate sensor calibration errors by automatically adjusting the data based on local gravity (van Hees et al., 2014) and calculating the Euclidean norm minus one (ENMO) for accelerations.

Sleep behavior was assessed as previously described by Valero-Cantero et al. (2021), involving the measurement of sustained inactivity periods (≤57 miliGravities, mG) with low z-angle variability (<5° over 5 min). A computer algorithm was then used to detect sleep onset and offset based on sustained inactivity periods (van Hees et al., 2018). Bedtime was defined as the estimated time participants went to bed, while sleep time was estimated as the difference between sleep onset and wake-up time. Wake after sleep onset (WASO) was defined as the total time spent awake between sleep onset and termination, while awakenings were defined as the number of times a person was awake for at least 5 min during the sleep period. Sleep efficiency was defined as the proportion of time spent sleeping from onset to termination, with a score of 100 indicating no waking occurred between sleep onset and termination.

To categorize physical activity and inactivity, pre-established ENMO thresholds for the wrist were utilized for older adults: (i) Inactivity: ENMO ≤57 mG, (ii) Light-intensity Physical Activity (LPA): ENMO >57 mG and <104 mG, and (iii) Moderate-to-Vigorous-intensity Physical Activity (MVPA): ENMO ≥104 mG (Sanders et al., 2019).

### 2.7. Statistical analyses

Categorical variables are presented as counts and percentages, while continuous variables are expressed as mean ± standard deviation (SD). The normality of continuous variables was assessed using the Kolmogorov–Smirnov test. The study sample was characterized through descriptive analyses. Additionally, the differences between physical frail and cognitive frail participants were assessed using Student’s *t*-test and chi-square test. To control for the potential impact of age on these differences, an Analysis of Covariance (ANCOVA) was conducted, with age utilized as a covariate.

Logistic regression models were utilized to explore the relationship between cognitive frailty and health factors. The models examined the associations between cognitive frailty status and variables such as physical frailty status, nutritional status, physical function, quality of life, sleep, and physical activity. An unadjusted model was used to test the initial associations between the variables. Variables that displayed significant values in Table 1 were included in the multivariable logistic regression models to identify potential confounders. Age was identified as the only significant confounder and was included in the adjusted model. Finally, odds ratio (95% confidence interval) and *p*-values were reported in each model.

**Table 1:** Participant characteristics by cognitive status.

	Total (*n* = 233)	Frailty (*n* = 178)	Cognitive frailty (*n* = 55)	*p*	Cognitive frailty OR (95% CI)	*p*
**Sex, *n* (%)**						
Men	77 (33.0)	64 (36.0)	13 (23.6)	0.090	Ref	0.092
Women	156 (67.0)	114 (64.0)	42 (76.4)		1.814 (0.907–3.628)	
Age (years)	74.82 ± 6.38	74.11 ± 6.08	77.13 ± 6.81	**0.002**	**1.077 (1.025–1.131)**	**0.003**
**Marital status, *n* (%)**						
Single	8 (3.4)	8 (4.5)	0 (0)	0.101	Ref	
Married	112 (48.1)	90 (50.6)	22 (40.0)		0.458 (0.048–4.420)	0.5
Widowed	79 (33.9)	54 (45.5)	25 (33.9)		1.426 (0.523–3.887)	0.488
Divorced	34 (14.6)	26 (14.5)	8 (14.6)		1.467 (0.524–4.105)	0.466
**Housing, *n* (%)**						
Not alone	144 (61.8)	114 (64.0)	30 (54.5)	0.419	Ref	
Alone	89 (38.2)	64 (36.0)	25 (45.5)		1.292 (0.693–2.410)	0.42
**Education status, *n* (%)**						
Less than primary school	47 (20.2)	32 (27.3)	15 (20.2)	0.073	2.585 (0.809–8.262)	0.109
Primary School	105 (45.1)	77 (50.9)	28 (45.1)		1.745 (0.607–5.019)	0.301
Secondary school	52 (22.3)	45 (12.7)	7 (22.3)		0.747 (0.214–2.606)	0.640
University and above	29 (12.4)	24 (9.1)	5 (12.4)		Ref	
Number of daily medications (number)	5.39 ± 3.85	5.20 ± 3.79	5.98 ± 4.00	0.195	1.053 (0.974–1.138)	0.195
**Number of falls in the last year, *n* (%)**						
None	152 (65.2)	124 (69.7)	28 (50.9)	**0.018**	Ref	
1–5	66 (28.3)	46 (25.8)	20 (36.4)		**2.111 (1.044–4.270)**	**0.038**
5–10	11 (4.7)	7 (3.9)	4 (7.3)		1.407 (0.141–14.062)	0.771
More than 10	4 (1.7)	1 (0.6)	3 (5.5)		**12.667 (1.268–126.557)**	**0.031**
**Need for walking assistance, *n* (%)**						
No	195 (83.7)	154 (74.5)	41 (74.5)	**0.042**	Ref	
Yes	38 (16.3)	24 (13.5)	14 (25.5)		**2.144 (1.017–4.521)**	**0.045**
**Monthly income, *n* (%)**						
Lower than 600 €	33 (23.7)	25 (23.8)	8 (23.5)	0.664	1.222 (0.433–3.445)	0.705
600–1,200 €	53 (38.1)	38 (36.2)	15 (44.1)		1.507 (0.617–3.682)	0.368
Higher than 1,200 €	53 (38.1)	42 (40.0)	11 (32.)		Ref	

Values are expressed as counts (percentages) or mean ± standard deviation, statistically significant values (*p* < 0.05) are bolded.

All analyses were performed by using the IBM SPSS Statistics 26 software (SPSS Inc., Chicago, IL, United States) and GraphPad Prism 9 (GraphPad Software, Boston, MA, United States), with a significance set at *p* < 0.05.

## 3. Results

The characteristics of the study sample are presented in Table 1, indicating that 23.61% of participants exhibited cognitive frailty. Cognitive frailty was significantly associated with advanced age, a higher occurrence of falls in the preceding year, and dependence on a mobility device for walking. Although all three variables were indicative of cognitive frailty, only age remained a statistically significant predictor when all three variables were incorporated into the same model.

Table 2 displays the outcomes concerning Fried’s frailty phenotype and cognitive frailty, indicating that individuals with cognitive frailty were more likely to experience exhaustion during the week and demostrate poorer performance in both the 4.57-meter gait and handgrip tests, when compared to those without cognitive frailty. After adjusting for age, expressing exhaustion on any day of the week and experiencing fatigue for more than 3 days per week were idenfified as significant risk factors for cognitive frailty, while performance in the handgrip strength test remained as a protective factor.

**Table 2:** Fried’s frailty characteristics by cognitive status and odds ratio for cognitive frailty.

	Total (*n* = 233)	Frailty (*n* = 178)	Cognitive frailty (*n* = 55)	*p*	Cognitive frailty OR (95% CI)	*p*	Adjusted Cognitive frailty OR (95% CI)	*p*
**Unintended weight loss, *n* (%)**								
Lost <5% of body mass	160 (68.7)	124 (69.7)	36 (68.7)	0.645	Ref	0.645	Ref	0.765
Lost >5% of body mass	73 (31.3)	54 (30.3)	19 (31.3)		1.163 (0.612–2.210)		1.107 (0.567–2.164)	
**Self-reported exhaustion and fatigue, *n* (%)**								
Did not meet the criteria	67 (28.8)	54 (30.3)	13 (23.6)	0.288	1.593 (0.757–3.350)	0.220	1.673 (0.783–3.575)	0.184
Met the criteria	166 (71.2)	124 (69.7)	42 (76.4)					
**Exhaustion, *n* (%)**								
Never	42 (18.0)	38 (21.3)	4 (7.3)	**0.041**	Ref		Ref	
1–2 days	42 (18.0)	31 (17.4)	11 (20.0)		**4.138 (1.041–16.444)**	**0.044**	**4.495 (1.109–18.224)**	**0.035**
3–4 days	69 (29.6)	53 (29.8)	16 (29.1)		**3.529 (1.001–13.093)**	**0.049**	**3.806 (1.008–14.370)**	**0.049**
5–7 days	80 (34.3)	56 (31.5)	24 (43.6)		**5.647 (1.580–20.185)**	**0.008**	**5.791 (1.594–21.033)**	**0.008**
**Fatigue, *n* (%)**								
Never	69 (29.6)	58 (32.6)	11 (20.0)	0.213	Ref		Ref	
1–2 days	60 (25.8)	46 (25.8)	14 (25.5)		1.702 (0.680–4.259)	0.255	1.981 (0.769–5.105)	0.157
3–4 days	49 (21.0)	35 (19.7)	14 (25.5)		2.234 (0.879–5.677)	0.091	**2.690 (1.021–7.085)**	**0.045**
5–7 days	55 (23.6)	39 (21.9)	16 (29.1)		2.316 (0.949–5.649)	0.065	**2.857 (1.130–7.229)**	**0.027**
**Physical activity expenditure, *n* (%)**								
Low	35 (15.0)	30 (16.9)	5 (9.1)	0.151	0.525 (0.189–1.496)	0.217	0.525 (1.189–1.461)	0.217
Normal	198 (85.0)	148 (83.1)	50 (90.9)					
Physical activity expenditure (kcal/week)	3031.62 ± 3173.28	3171.45 ± 3324.75	2584.18 ± 2607.46	0.232	1.000 (1.000–1.000)	0.29	1.000 (1.000–1.000)	0.502
**Gait speed, *n* (%)**								
Low	61 (26.2)	42 (23.6)	19 (26.2)	0.140	1.580 (0.810–3.085)	0.180	1.213 (0.598–2.461)	0.593
Normal	172 (73.8)	136 (65.5)	36 (73.8)					
4.57-meter Gait test (s)	6.02 ± 2.52	5.79 ± 2.41	6.73 ± 2.75	**0.016**	**1.131 (1.009–1.268)**	**0.034**	1.068 (0.945–1.208)	0.293
**Handgrip strength, *n* (%)**								
Low	143 (61.4)	105 (59.0)	38 (69.1)	0.095	1.824 (0.903–3.685)	0.094	1.526 (0.740–3.147)	0.252
Normal	90 (38.6)	73 (41.0)	17 (30.9)					
Handgrip strength (kg)	21.51 ± 9.45	22.31 ± 9.80	18.80 ± 7.64	**0.019**	**0.954 (0.919–0.991)**	**0.014**	**0.963 (0.927–0.999)**	**0.049**
**Frailty status, *n* (%)**								
Pre-frail	161 (69.1)	129 (58.2)	32 (69.1)	**0.033**	**1.928 (1.014–3.666)**	**0.045**	1.600 (0.819–3.127)	0.169
Frail	72 (30.9)	49 (41.8)	23 (30.9)					
Frail criteria	2.05 ± 0.97	1.99 ± 0.95	2.24 ± 1.01	0.098	**1.294 (0.950–1.763)**	**0.103**	1.187 (0.860–1.639)	0.297

Values are expressed as counts (percentages) or mean ± standard deviation. Statistically significant values (*p* < 0.05) are bolded. OR, Odd Ratio. Adjusted OR ratio included age as covariable.

**Figure 1:**
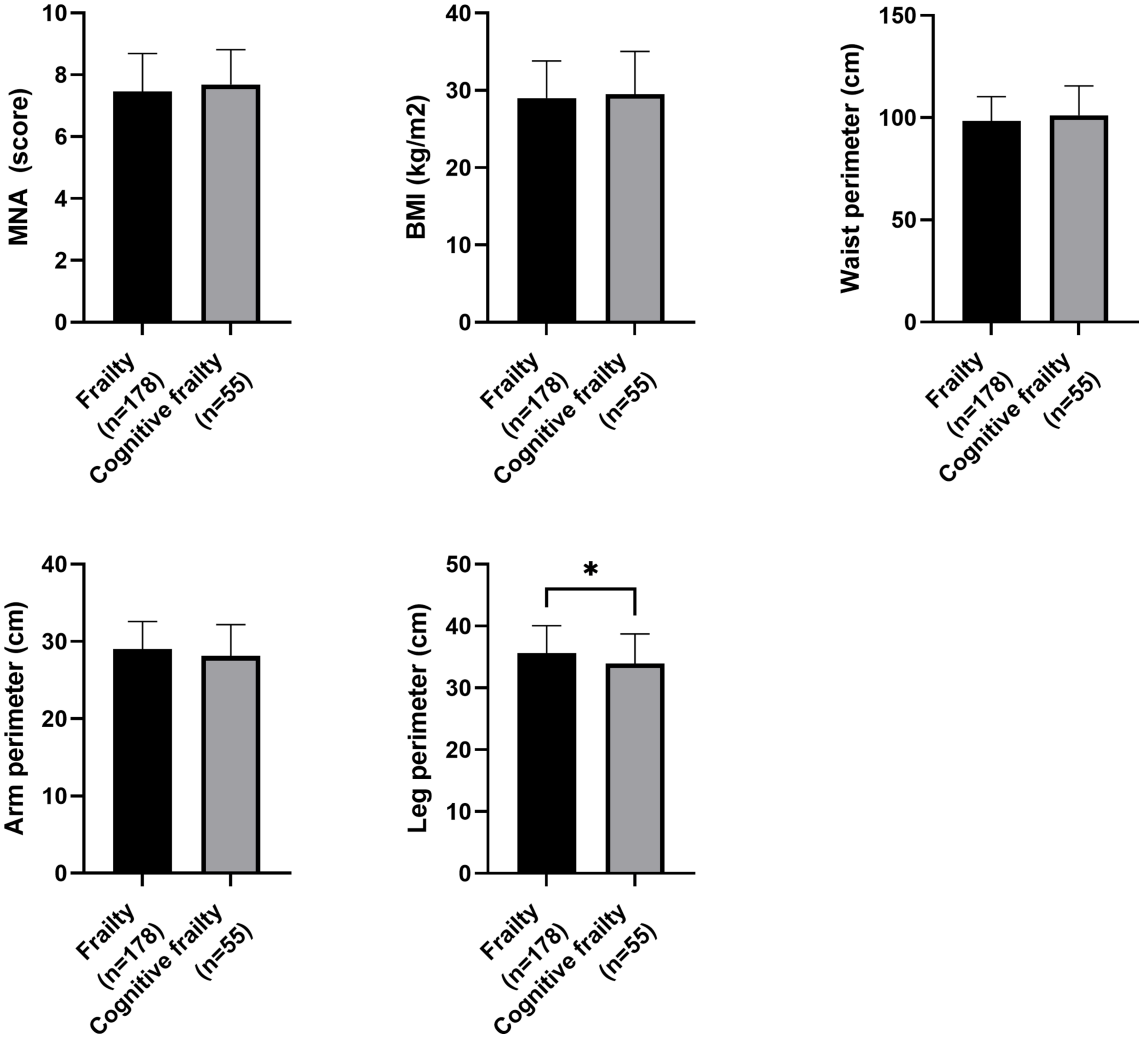
Differences in nutritional status between frail and cognitive frail participants. MNA, Mini Nutritional Assessment; BMI, Body Mass Index; *p < 0.033.

**Figure 2:**
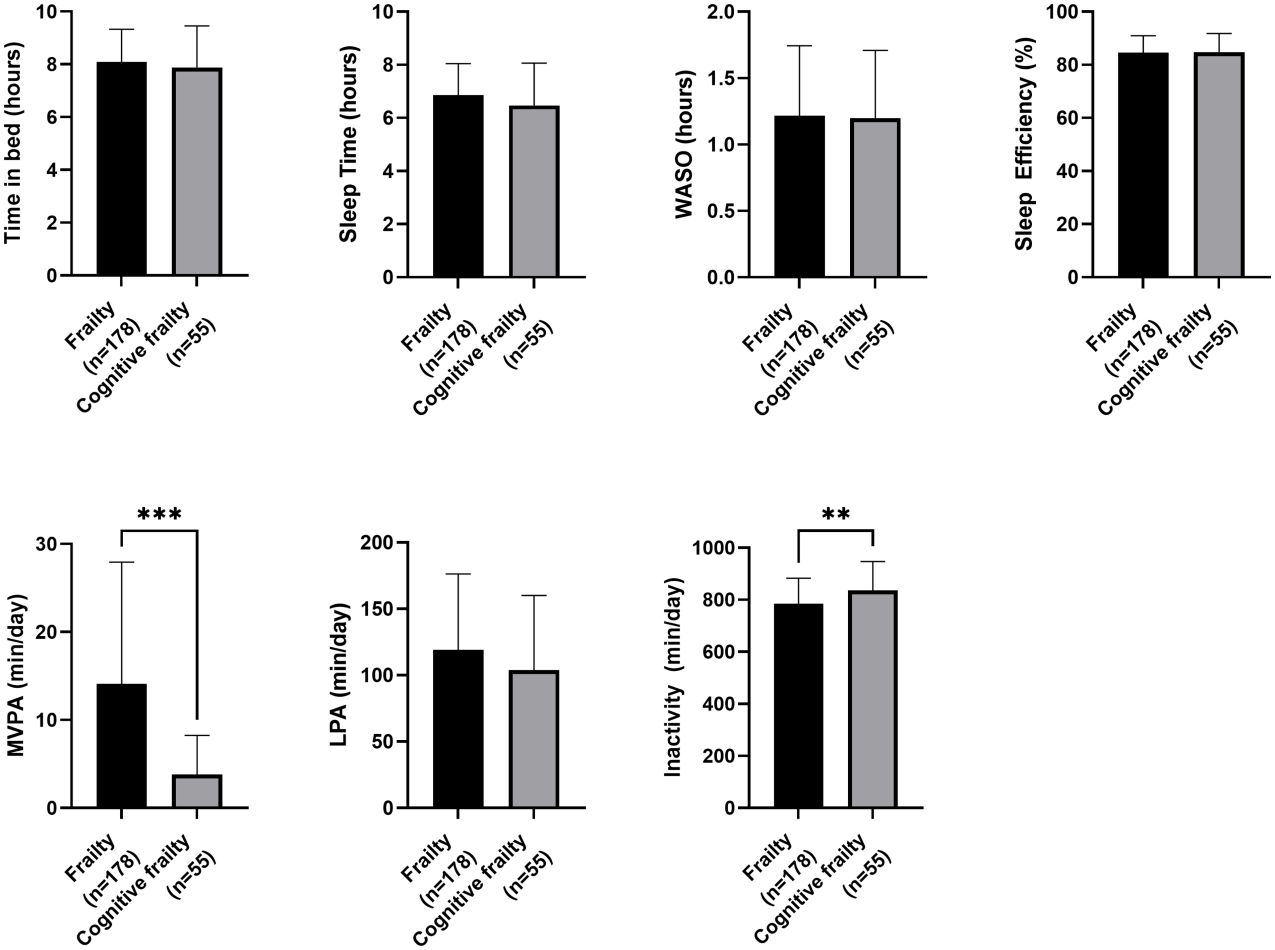
Differences in physical function and quality of life between frail and cognitive frail participants. STS, Sit-to-Stand test; SPPB, Short Physical Performance Battery; QoL, Quality of life; VAS, Visual-Analogue Scale; *p < 0.033.

**Figure 3:**
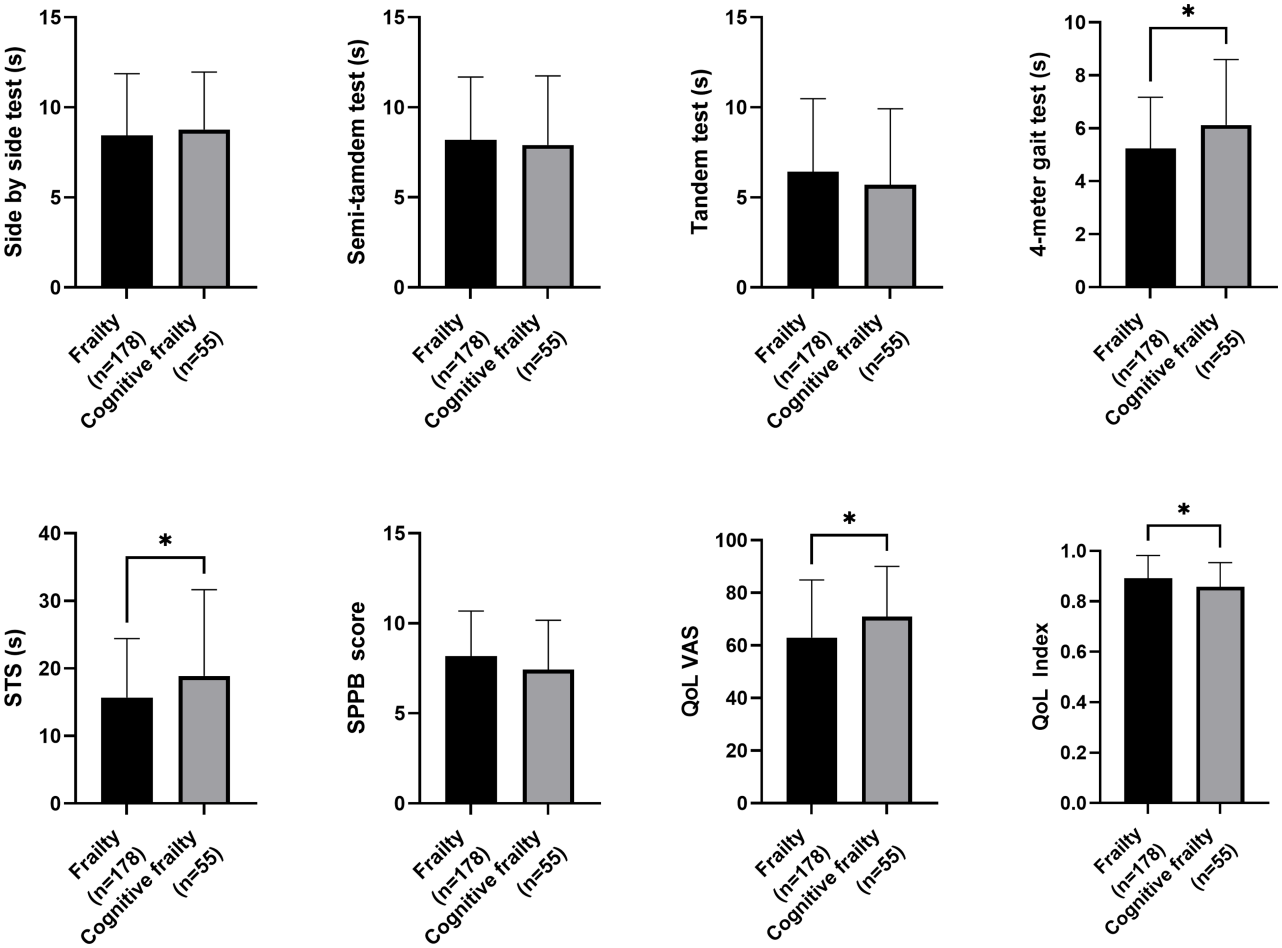
Differences in sleep behavior and physical activity between frail and cognitive frail participants. WASO, Wake After Sleep Onset; MVPA, Moderate-to-Vigorous-intensity Physical Activity; LPA, Light-intentisy Physical Activity; *p < 0.033; **p < 0.002.

Regarding nutritional status, participants with cognitive frailty had reduced values for leg perimeter (as depicted in Figure 1). Concerning physical function and quality of life, participants with cognitive frailty demonstrated poorer performance in the 4-meter gait and sit-to-stand tests and, although they obtained higher scores on the QoL-VAS, significantly lower EQoL-Index scores were observed (as shown in Figure 2). In terms of sleep behavior and daily levels of physical activity and inactivity, participants with cognitive frailty had higher inactivity time and lower levels of MVPA compared to their counterparts without cognitive frailty (as shown in Figure 3).

In logistic regression models, all of the aforementioned variables that showed significant differences between the groups were also significantly associated with the risk of cognitive frailty, except for the sit-to-stand test and inactivity time (as reported in Table 3). After adjusting for age, QoL-VAS was identified as a risk factor for cognitive frailty, whereas calf perimeter, EQoL-Index, and time spent in MVPA were identified as protective factors.

**Table 3:** Nutritional status, physical function, sleep, and physical activity odds ratios for cognitive frailty.

	Cognitive frailty OR (95% CI)	*p*	Adjusted cognitive frailty OR (95% CI)	*p*
**Nutritional status**				
MNA (score)	1.183 (0.916–1.527)	0.197	1.188 (0.913–1.546)	0.200
BMI (kg/m^2^)	1.084 (0.960–1.084)	0.515	1.063 (0.993–1.137)	0.078
Waist perimeter (cm)	1.017 (0.993–1.041)	0.160	1.015 (0.991–1.040)	0.209
Arm perimeter (cm)	0.976 (0.903–1.055)	0.541	0.989 (0.911–1.072)	0.782
Calf perimeter (cm)	**0.914 (0.849–0.984)**	**0.017**	**0.929 (0.863–0.999)**	**0.049**
**Physical function and quality of life**				
Side-by-side test (s)	1.046 (0.945–1.159)	0.384	1.078 (0.970–1.197)	0.162
Semitandem test (s)	0.987 (0.906–1.075)	0.764	1.020 (0.932–1.116)	0.674
Tandem test (s)	0.954 (0.886–1.028)	0.220	0.983 (0.909–1.062)	0.660
Sit-to-stand test (s)	1.004 (0.988–1.021)	0.594	1.000 (0.983–1.017)	0.963
4-meter gait test (s)	**1.180 (1.025–1.358)**	**0.021**	1.132 (0.979–1.309)	0.094
SPPB score (score)	0.895 (0.794–1.008)	0.066	0.944 (0.831–1.072)	0.376
QoL-VAS (score)	**1.021 (1.003–1.039)**	**0.023**	**1.024 (1.004–1.044)**	**0.015**
EQoL-index (score)	**0.027 (0.001–0.775)**	**0.035**	**0.025 (0.001–0.771)**	**0.035**
**Sleep and physical activity**				
Time in bed (hours)	0.890 (0.699–1.132)	0.343	0.860 (0.674–1.098)	0.226
Sleep time (hours)	0.808 (0.629–1.038)	0.096	0.794 (0.614–1.022)	0.074
WASO (hours)	1.266 (0.837–1.915)	0.265	1.207 (0.791–1.840)	0.383
Sleep efficiency (%)	2.072 (0.023–187.101)	0.751	4.938 (0.054–455.780)	0.489
MVPA (min/day)	**0.969 (0.946–0.993)**	**0.012**	**0.973 (0.949–0.997)**	**0.029**
LPA (min/day)	1.004 (0.998–1.011)	0.189	1.005 (0.998–1.011)	0.181
Inactivity time (min/day)	1.000 (0.996–1.003)	0.843	1.000 (0.996–1.003)	0.794

Adjusted OR ratio included age as a covariable. Statistically significant values (*p* < 0.05) are bolded. OR, Odd Ratio; MNA, Mininutritional Assessment; BMI, Body Mass Index; SPPB, Short-Performance Physical Battery; QoL-VAS, Quality of life Visual-Analogue Scale; EQoL-Index, Quality of Life EuroQol Index; WASO, Wake After Sleep Onset; MVPA, Moderate-to-Vigorous-intensity Physical Activity; LPA, Light-intensity Physical Activity.

## 4. Discussion

In this multicentre cross-sectional study, we enrolled 233 community-dwelling older adults who met Fried’s criteria for frailty phenotype. Our sample showed a cognitive frailty prevalence of 24%, which is comparable to findings in a prior study (Katayama et al., 2021). The influence of sex on the development of cognitive frailty remains an unresolved question. In our sample, consisting of a higher proportion of women (67%) than men (33%), there was no significant difference in the prevalence of cognitive frailty between sexes. Moreover, the logistic regression analyses did not identify sex as a risk factor for this condition.

These findings are in line with previous research that has not identified sex as an independent risk factor for cognitive frailty (Ruan et al., 2020). Furthermore, a systematic review and meta-analysis of previous studies revealed a lack of association between sex and cognitive frailty (Zhang et al., 2022). Our findings add to this evidence and suggest that sex may not have a significant influence on the development of cognitive frailty in older adults with frailty phenotype.

Numerous sociodemographic variables have been associated with the development of cognitive frailty. Advancing age is a well-known risk factor for developing frailty and cognitive frailty, as supported by robust scientific evidence (Navarro-Pardo et al., 2020; Katayama et al., 2021). Consistent with these findings, our study identified age as a significant and independent risk factor for cognitive frailty in frail and pre-frail older adults living in the community.

Falls represent another condition commonly linked to cognitive frailty. Our study revealed that experiencing falls between 1 and 5 times, as well as more than 5 times within the last year, were identified as risk factors for cognitive fraity. This is in line with previous data that demonstrated that cognitively impaired adults are at higher risk of falling than those without cognitive impairment (Tsutsumimoto et al., 2018). The need for walking assistance was also identified as a sociodemographic variable that increases the risk of cognitive frailty, which is similar to previous studies demonstrating an association between cognitive frailty and disability (Panza et al., 2018).

However, after conducting a logistic regression analysis including all sociodemographic variables, age was the only variable that remained a significant risk factor. Given the fact that previous research has identified age as a primary risk factor for these conditions (Stewart Williams et al., 2015), these results suggest that in older people with frailty phenotype, age is the main variable contributing to the development of cognitive frailty, falls, and disability. Therefore, we included age as an adjusting variable in our analyses to account for its potential influence on the relationship between other health factors and cognitive frailty.

Our findings revealed that the proportion of older adults with cognitive frailty who reported exhaustion and fatigue was similar to those without cognitivefrailty (76.4% vs. 69.7%). However, reporting exhaustion for any day of the week and fatigue for more than 3 days per week were identified as significant risk factors for the development of cognitive frailty. These results emphasize the importance of considering psychological factors such as exhaustion and fatigue as mediators in the relationship between frailty and cognition (Robertson et al., 2013, 2014).

Thus, cognitive frailty has been consistently associated with a higher risk of poor quality of life (Arai et al., 2018). Interestingly, our study yielded contrasting results when evaluating the quality of life in our population. While the self-perceived quality of life, assessed using a QoL-VAS, emerged as a significant risk factor for cognitive frailty in both unadjusted and adjusted models, the EQoL-Index, which encompasses responses to various daily life activities, was found to be a protective factor. These findings suggest that using the complete version of the quality of life tool is essential.

Although utilizing only the QoL-VAS scale may appear convenient for a shorter evaluation time, it may not be appropriate for individuals with frailty or pre-frailty and cognitive impairment. Notably, our results indicate that participants with cognitive frailty tend to have a more favorable perception of their health status compared to those with frailty alone. These findings shed light on the complex relationship between cognitive frailty and quality of life, highlighting the importance of utilizing comprehensive measures to capture the multidimensional aspects of well-being.

Compelling evidence highlighting a significant interconnection between physical frailty and cognitive decline, specifically emphasizing executive dysfunction, has been reported (Amanzio et al., 2017; Bartoli et al., 2020). The findings underscore the intricate interplay between the physiological manifestations of physical frailty and the cognitive intricacies of neurodegenerative conditions, shedding light on potential shared mechanisms and pathways that contribute to the complex clinical landscape of these disorders. Although we excluded neurocognitive disorders in our study, our findings are in line with this relationship between physical and cognitive frailty.

Specifically, our results demonstrated that handgrip strength was a significant protective factor against cognitive frailty. These findings align with previous cross-sectional and prospective studies, providing strong evidence that physical health, as indicated by handgrip strength, is associated with cognitive function in older adults over time (Luo et al., 2022). Regarding gait speed, although the time spent in the 4.57-meter gait test was initially associated with cognitive frailty, this association became non-significant after adjusting for age. The same pattern occurs with the evaluation of the gait speed of the SPPB, which was the only variable of the battery that was associated with cognitive frailty. Thus, age might be a more influential factor for cognitive impairment than gait speed.

This finding partially contradicts previous studies that supports the motoric cognitive risk syndrome (Verghese et al., 2013) and that identified gait speed as a significant predictor of cognitive frailty (Armstrong et al., 2020). The motoric cognitive risk syndrome, which has several subtypes according to individual quantitative measures of cognitive impairment (Bortone et al., 2022), has been associated with sarcopenia, body fat indices and systemic inflammation in pre-frail older adults (Merchant et al., 2023). As a result, these observations prompt further investigation and exploration into the complex interplay of these factors in the context of cognitive frailty.

Prior research has demonstrated a connection between cognitive decline in older adults, particularly those experiencing motoric cognitive risk syndrome, and reduced levels of physical activity (Bortone et al., 2021). Aligning with these findings, our study revealed notable differences in the daily levels of MVPA and inactivity between participants with and witouth cognitive frailty. Cognitive frail individuals spent significantly less time engaged in MVPA and more time in inactive behaviors compared to their counterparts witouth cognitive frailty. Importantly, only MVPA was found to be significantly associated with a reduced risk of cognitive frailty.

These findings contribute to the growing body of evidence supporting the beneficial effects of physical activity, particularly at intensities higher than moderate, in the management of cognitive frailty. Similar positive effects of increasing MVPA levels on cognitive frailty have been observed in both shorter (12 weeks) (Kwan et al., 2020) and longer interventions (24 months) (Liu et al., 2018). These findings underscore the importance of incorporating progressively regular and sustained MVPA into the daily-life style of older adults with frailty with or without cognitive impairment at preventing and managing cognitive frailty in older adults.

Although participants with cognitive frailty demonstrated a longer time to complete 5 repetitions of the sit-to-stand test compared to those without cognitive frailty, this variable did not show a significant association with this condition. These findings suggest that while the sit-to-stand test is commonly used to evaluate overall physical function (Yoon et al., 2018), the strength and function of the knee extensor/flexor muscles may not be a determining factor for cognitive impairment in older adults with frailty. This is in contrast with the observed utility of accelerometers in assessing daily levels of physical activity of frail or pre-frail older adults living in the community, where the significance of wearable sensors has been underscored (Zhou et al., 2019; Razjouyan et al., 2020).

Consistent with this, among the nutritional status variables investigated in our study, we identified calf circumference as a notable protective factor against the development of cognitive frailty. These findings align with previous research that has demonstrated an inverse association between calf circumference and disability, specifically highlighting the importance of leg circumference (Sun et al., 2017). This suggests that calf circumference, serving as a simple proxy for skeletal muscle mass and sarcopenia, may play a crucial role in the development of frailty and cognitive impairment. Reduced calf circumference is indicative of muscle mass atrophy, which has been identified as a significant factor in the pathogenesis of both frailty and cognitive impairment (Kim et al., 2018).

The significant association between calf circumference and cognitive frailty underscores the potential utility of measuring calf circumference as a means of monitoring cognitive frailty in older adults with frailty. This measurement could prove valuable in both clinical and research settings, providing a practical and accessible tool for assessing muscle mass and its implications for cognitive health. Further investigation is warranted to explore the underlying mechanisms linking calf circumference, skeletal muscle mass, and cognitive frailty, which may pave the way for targeted interventions aimed at preserving muscle mass and mitigating the risk of cognitive decline in older individuals with frailty.

The risk of malnutrition assessed using the MNA is a viable tool for monitoring changes in older adults (Casals et al., 2015). Previous research has shown that the MNA score in frail or pre-frail older adults is linked to physical function and independency and has been suggested as a predictor of frailty (Casals-Vázquez et al., 2017; Casals et al., 2018; Kiljunen et al., 2023). Nevertheless, this association was absent in our sample, thus, a further dietary assessment is encouraged in future studies.

It is important to acknowledge several limitations associated with our multicenter cross-sectional study conducted among community-dwelling frail and pre-frail older adults. The cross-sectional design restricts our ability to establish causal relationships or track changes in variables over time. The findings should be interpreted as associations rather than causation. Also, there may be a potential for selection bias, as our study only included individuals residing in the community, excluding those who are institutionalized or have more advanced health conditions, such as dementia or Alzheimer’s disease. This may limit the generalizability of our results to broader populations. Additionally, the sample size of our study should be considered. While we made efforts to include an adequate number of participants, a larger sample size would enhance the statistical power and generalizability of the findings.

Furthermore, it is imperative to underscore that the cognitive status of the participants was exclusively evaluated using a screening test, a method that can be readily administered by nurses in clinical care settings. Notwithstanding this advantage, this approach may have limitations in fully capturing the participants’ cognitive abilities. Hence, future studies incorporating a more comprehensive set of neuropsychological tests are warranted to provide a more thorough evaluation of these associations. Similarly, the assessment of the nutritional status encompassed easily accessible measures (BMI, body circumferences, and the MNA) to ensure its unfeasibility for routine clinical implementation. However, the omission of a more comprehensive analysis of dietary intake highlights the need for a more thorough evaluation of participants’ eating habits.

Despite these limitations, our study provides valuable insights into the characteristics of frail and pre-frail older adults living in the community and their risk of cognitive frailty. By identifying these associations, we contribute to the existing body of knowledge and pave the way for future research in this field. It is crucial to consider these limitations when interpreting the findings and to further investigate these relationships in longitudinal studies with larger and more diverse populations.

## 5. Conclusion

In conclusion, our study highlights the complex interplay of both non-modifiable and modifiable factors in cognitive frailty among older adults with a frailty phenotype living in the community. Age remains a key non-modifiable factor associated with cognitive impairment in this population. However, our findings also shed light on several modifiable factors that can be targeted for intervention.

Nutritional status, quality of life, and physical activity are identified as important modifiable factors associated with cognitive frailty. Specifically, our results suggest that assessing calf circumference may provide valuable insights into cognitive risk factors in older adults with frailty, surpassing the utility of other commonly used anthropometric measures. Furthermore, daily levels of moderate-to-vigorous physical activity (MVPA) emerge as a protective factor against cognitive frailty, emphasizing the importance of incorporating regular MVPA into preventive and management strategies.

Additionally, our findings highlight the relevance of considering psychological outcomes, such as self-reported exhaustion and fatigue, alongside physical frailty indicators like handgrip strength, as risk factors for cognitive frailty. Fried’s criteria, without strict cut-off points, offer a comprehensive tool for evaluating both physical and psychological aspects of frailty.

In summary, this study underscores the need to address a range of modifiable factors in the prevention and management of cognitive frailty in older adults with a frailty phenotype. By targeting nutritional status, quality of life, and physical activity, and considering psychological factors, healthcare interventions can strive to mitigate the risk and impact of cognitive frailty on older individuals’ overall well-being and independence.

## Data availability statement

The raw data supporting the conclusions of this article will be made available by the authors, without undue reservation.

## Ethics statement

The studies involving humans were approved by Ethics Committee of Provincial Research of Málaga. The studies were conducted in accordance with the local legislation and institutional requirements. The participants provided their written informed consent to participate in this study.

## Author contributions

CC and MV-S participated in the research concept and study design. JC-P and CC did the literature review. JC-P, CC, LÁ-C-d-V, AG-M, IM-Z, FV-E, RR-C, and MV-S contributed to the data collection. JC-P, CC, and MV-S did the data analysis, interpretation, and statistical analysis. JC-P wrote the manuscript. CC, LÁ-C-d-V, AG-M, IM-Z, FV-E, RR-C, and MV-S reviewed and edited the manuscript. All authors contributed to the article and approved the submitted version.

## Funding

This study (project UMA20-FEDERJA-154) has been funded by 10.13039/501100011011 Junta de Andalucía and ERDF in the framework of the projects ERDF-Andalusia Operational Programme 2014–2020 (Programa Operativo FEDER Andalucía 2014–2020). LÁ-C-d-V and AG-M are supported by a research collaboration grant from the Spanish Ministry of Education and Vocational Training (Ministerio de Educación y Formación Profesional) (grant number 22CO1/012259 and 22CO1/009662, respectively).

## Conflict of interest

The authors declare that the research was conducted in the absence of any commercial or financial relationships that could be construed as a potential conflict of interest.

## Publisher’s note

All claims expressed in this article are solely those of the authors and do not necessarily represent those of their affiliated organizations, or those of the publisher, the editors and the reviewers. Any product that may be evaluated in this article, or claim that may be made by its manufacturer, is not guaranteed or endorsed by the publisher.
